# Recurrent Onychocryptosis Treated With the Winograd Procedure: A Case Report

**DOI:** 10.7759/cureus.104888

**Published:** 2026-03-09

**Authors:** Aaron D McCusker, Conn Rooney, Joshua Letch, Richard J Lang

**Affiliations:** 1 Podiatric Surgery, East Suffolk and North Essex NHS Foundation Trust, Ipswich, GBR; 2 Podiatric Surgery, Homerton Healthcare NHS Foundation Trust, London, GBR; 3 Podiatric Surgery, Buxton Hospital, Derbyshire Community Health Services, Buxton, GBR; 4 Podiatric Surgery, Essex Partnership University NHS Foundation Trust, Essex, GBR

**Keywords:** chemical matrixectomy, incisional nail surgery, onychocryptosis, partial nail avulsion, winograd procedure

## Abstract

Onychocryptosis is a common disorder of the nail unit in which the nail plate penetrates the surrounding periungual soft tissue. This often results in pain, inflammation, and potential infection. While conservative and non-incisional surgical treatments such as partial nail avulsion with chemical matrixectomy are often effective, recurrence may occur and can significantly impact the patient's quality of life. Chronic or recurrent cases may therefore require more definitive surgical management. We present the case of a 22-year-old male patient with recurrent onychocryptosis of the right hallux following failed partial nail avulsion and phenol matrixectomy. The patient subsequently underwent incisional nail surgery using the Winograd procedure. Postoperative recovery was uncomplicated with the patient returning to normal footwear within two weeks. At the 12-month follow-up, the hallux was fully healed with no evidence of nail regrowth or recurrence. The patient reported complete resolution of symptoms and was highly satisfied with the outcome. This case demonstrates that surgical excision with partial matrixectomy using the Winograd technique can provide a safe and effective long-term solution for recurrent onychocryptosis when less invasive interventions have failed.

## Introduction

Onychocryptosis, commonly referred to as an ingrowing toenail, is a frequent disorder of the nail unit in which the nail plate penetrates the surrounding periungual soft tissue, most often at the medial or lateral nail sulcus [1]. The condition is associated with pain, inflammation, and, in more advanced cases, infection and hypergranulation tissue formation. Heifetz designed a simple three-stage classification of ingrown toenails: stage 1 is swelling of the medial or lateral sulcus, stage 2 is acute infection, and stage 3 is chronic infection with hypergranulation tissue [2].

It is estimated that approximately 10,000 new cases are recorded annually in the United Kingdom, with young adult men most commonly affected. A number of predisposing factors have been identified, including genetic nail morphology, systemic conditions such as hyperhidrosis or onychauxis, inappropriate nail-cutting techniques, ill-fitting footwear, and obesity [3,4]. Management strategies depend on disease severity and typically begin with conservative measures such as nail bracing, packing, or taping. However, these approaches often provide only temporary symptom relief, particularly in moderate to severe or recurrent disease [5]. When conservative management fails, surgical intervention may be required. Non-incisional techniques, such as partial and total nail avulsion with chemical matrixectomy, are widely used. While the procedures generally demonstrate good outcomes, recurrence remains a recognized complication. In cases of chronic or recurrent onychocryptosis, incisional nail surgery techniques such as the Winograd procedure offer a more definitive treatment option by excising the affected nail matrix and hypertrophic soft tissue [1,4,5]. This case report describes the successful use of the Winograd procedure in a young adult man with recurrent onychocryptosis of the hallux following failed partial nail avulsion and chemical matrixectomy.

## Case presentation

**Figure 1:**
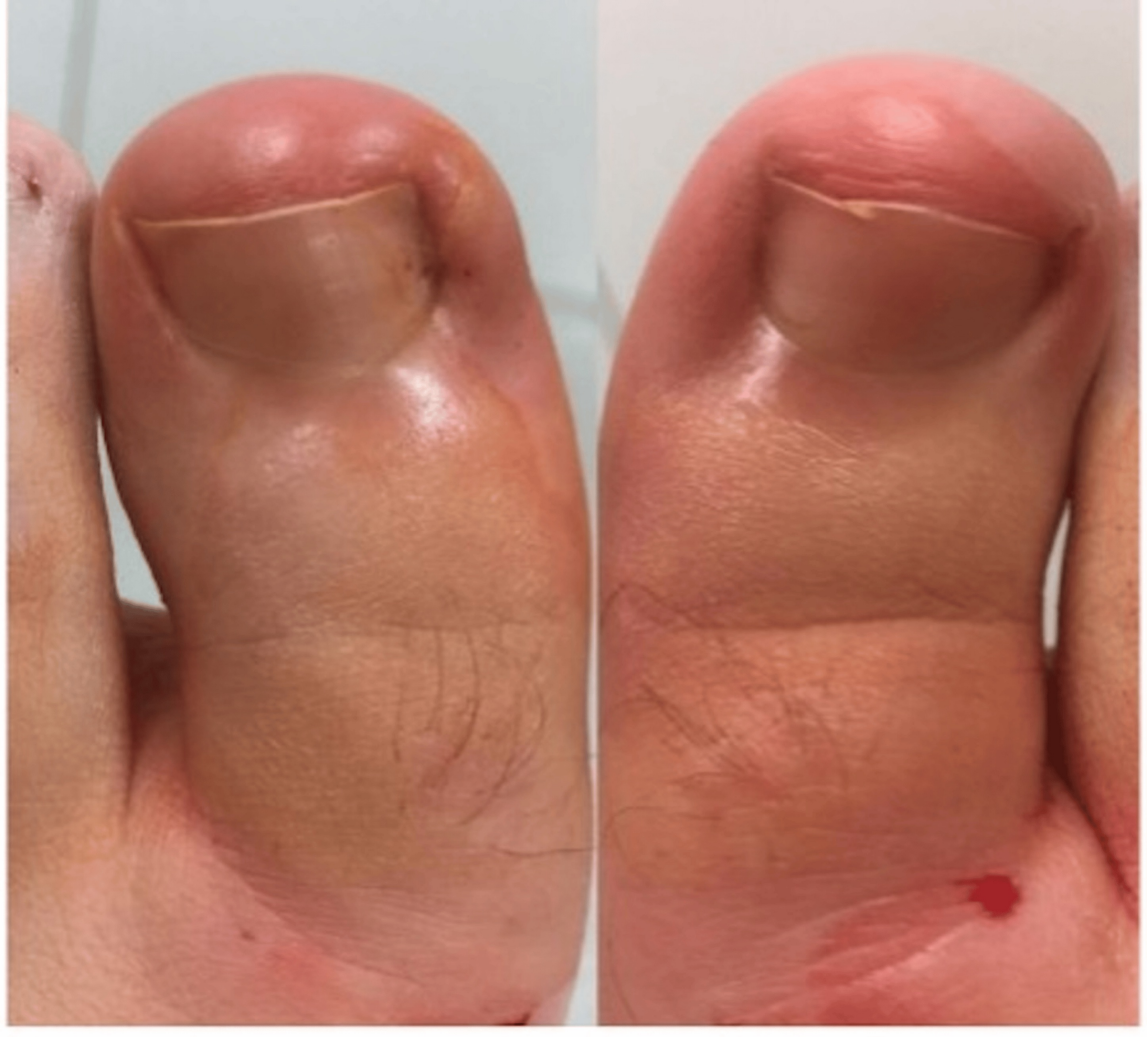
Initial presentation demonstrating bilateral onychocryptosis characterized by lateral nail plate impingement and localized periungual inflammation

A 22-year-old male patient presented to the Department of Foot and Ankle Surgery with a long-standing history of painful bilateral onychocryptosis affecting both halluces (Figure 1).

**Figure 2:**
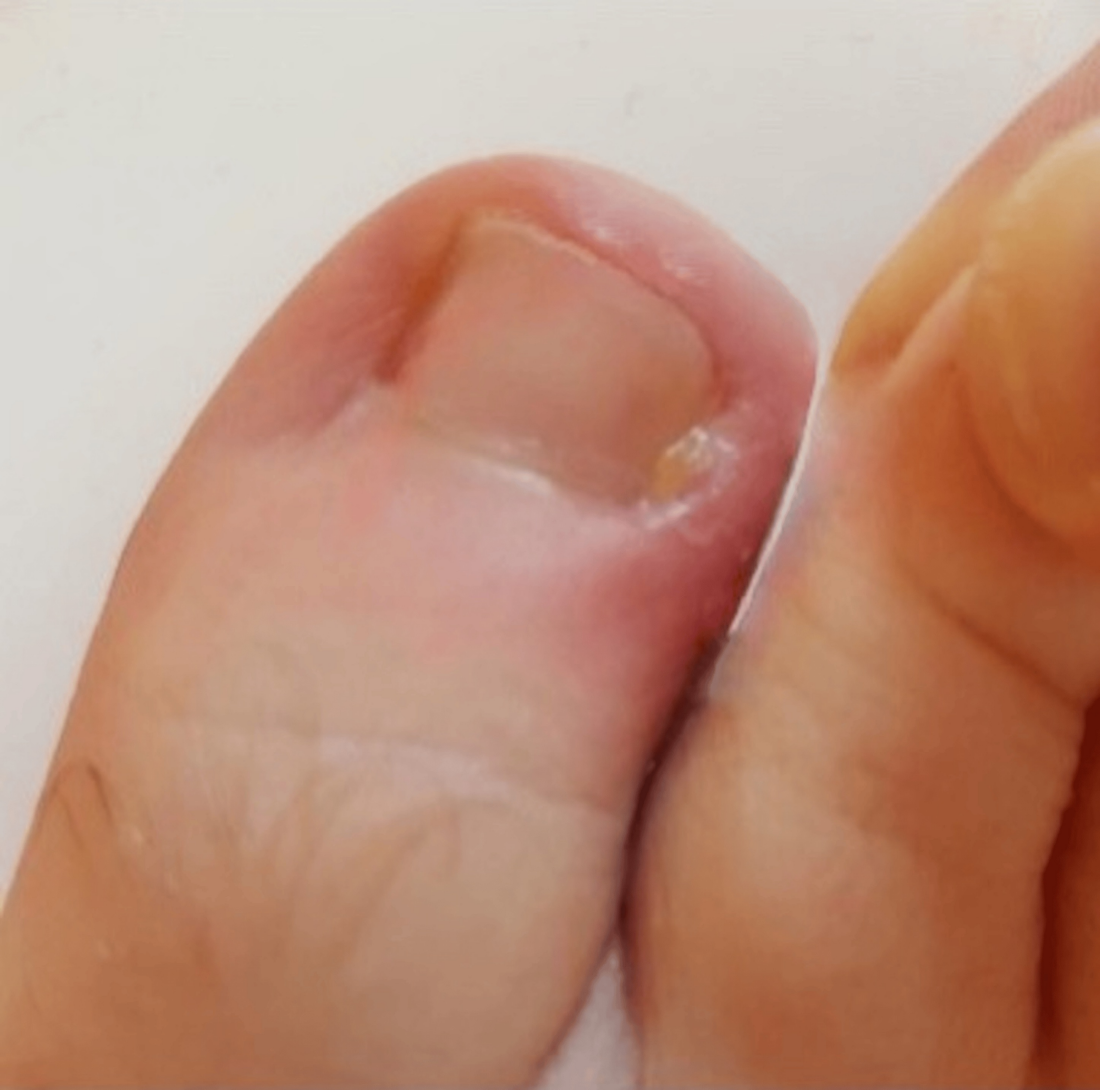
Clinical photograph demonstrating regrowth of the nail following partial nail avulsion with phenol matrixectomy. Notice the hypergranulation tissue with surrounding erythema

The condition had been present throughout adolescence and was previously managed conservatively with regular nail care and cotton wool packing, providing only temporary symptom relief. The patient was otherwise fit and well with no significant past medical history, no known allergies, and no history of smoking. Neurovascular examination of both feet was normal. The patient reported increasing pain, particularly after prolonged periods of standing during his work as a scrub practitioner. Although he described having developed a degree of pain tolerance, symptom severity had progressed to the point of significantly affecting daily activities and footwear tolerance. On clinical examination, the condition was consistent with moderate onychocryptosis (Heifetz stage II). Following failure of conservative management, the patient elected to undergo bilateral partial nail avulsions with chemical matrixectomy. The procedures were performed under digital nerve block anaesthesia with phenol applied to the nail matrices for four minutes. Postoperative recovery was initially uncomplicated, and the patient reported immediate symptomatic relief following the resolution of procedural discomfort. He remained compliant with postoperative care instructions. At 12 weeks postoperatively, the patient re-presented with recurrent pain affecting the lateral nail sulcus of the right hallux. Clinical examination revealed nail spicule regrowth with associated hypergranulation tissue, persistent serous discharge, maceration, malodour, and surrounding erythema (Figure 2).

The findings were consistent with severe recurrent onychocryptosis (Heifetz stage III). The left hallux remained asymptomatic and had completely healed. Given the recurrence following chemical matrixectomy, the patient expressed a preference for a treatment option offering the greatest likelihood of long-term symptom resolution. After discussing the surgical options, a Winograd procedure was recommended. Written informed consent was obtained, and the patient was scheduled for surgery in the podiatric surgery day case unit.

Surgical technique

**Figure 3:**
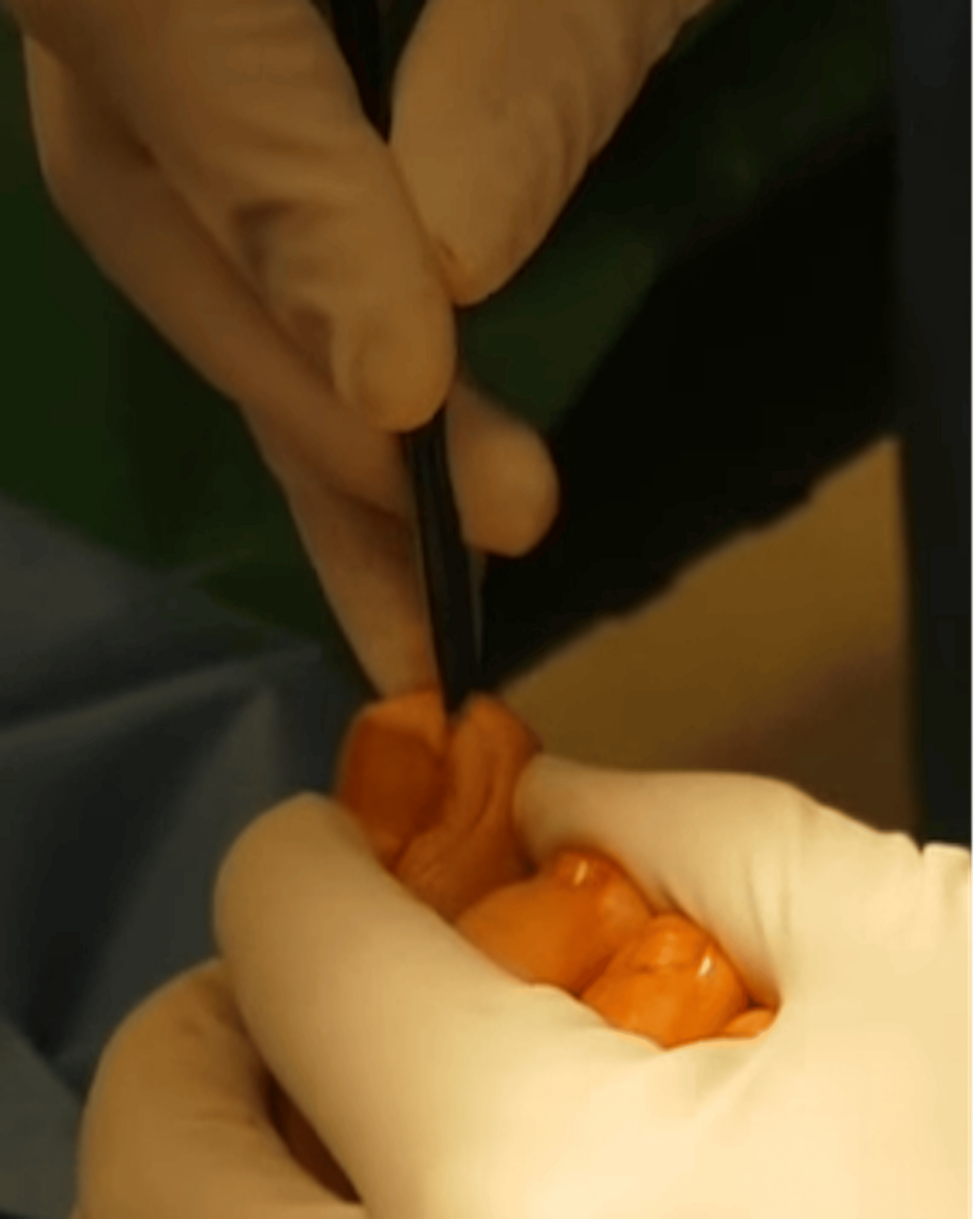
Intraoperative image during the Winograd procedure demonstrating the excision of the lateral nail fold, the involved nail plate segment, the hypergranulation tissue, and the germinal matrix

The patient was admitted under the care of a consultant podiatric surgeon. Following standard preoperative checks, the right foot was prepared in accordance with World Health Organization surgical safety standards. A popliteal nerve block was administered at the patient's request. The procedure was performed in a clean-air operating environment. A digital tourniquet was applied to achieve haemostasis. The hypertrophic periungual tissue of the lateral nail sulcus was excised using a Winograd incision. The lateral portion of the nail plate, nail matrix, and associated soft tissue were sharply excised down to the level of the periosteum using a No. 10 blade (Figure 3).

**Figure 4:**
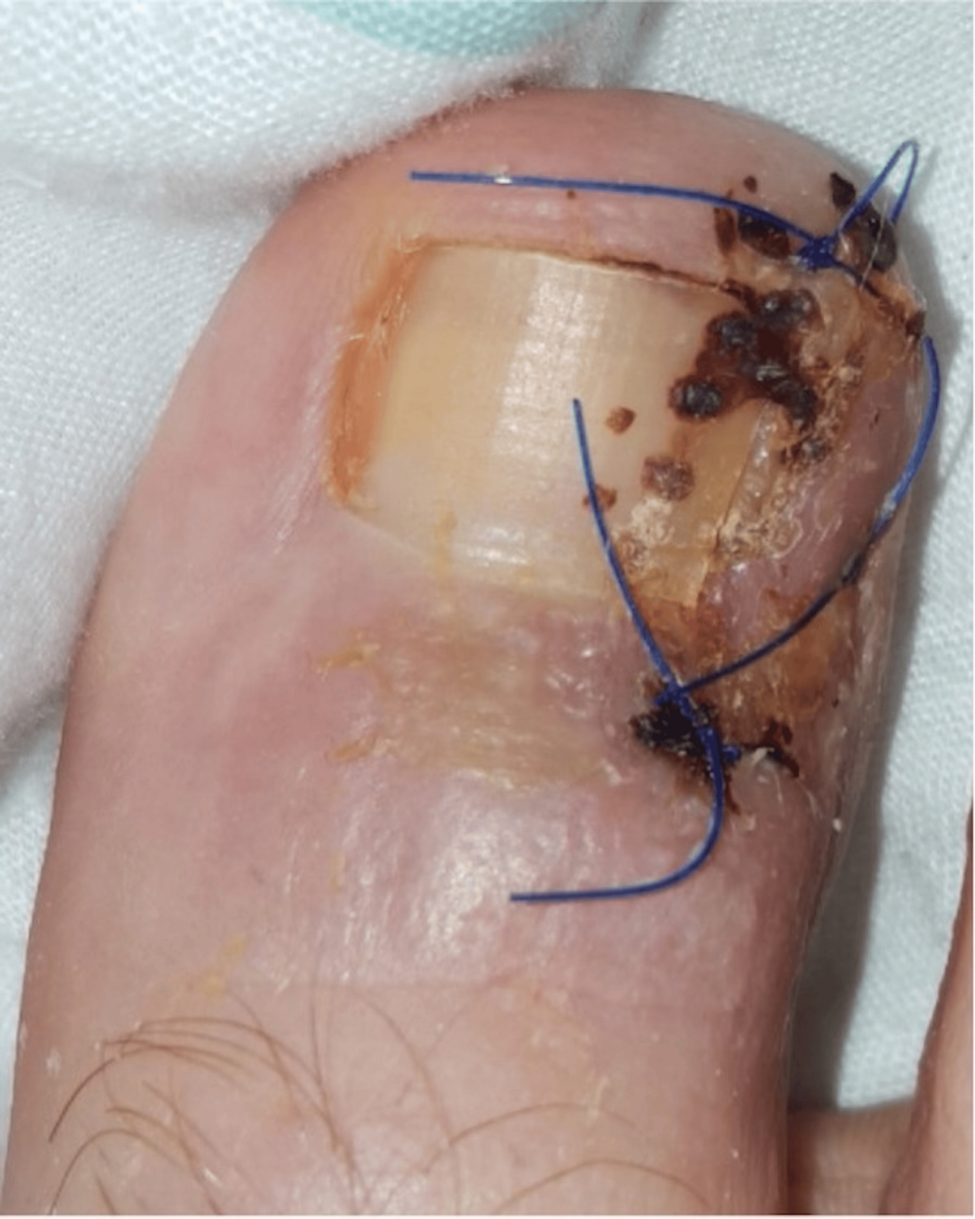
Two-week postoperative appearance following the Winograd procedure demonstrating satisfactory healing of the lateral nail fold

The wound was irrigated with saline and closed using 3-0 Prolene sutures. Upon release of the tourniquet, satisfactory vascular return was observed. A sterile dressing was applied, and a heel-rocker postoperative shoe was provided to offload the forefoot. At the one-week follow-up, the surgical site demonstrated satisfactory healing with no signs of infection (Figure 4).

**Figure 5:**
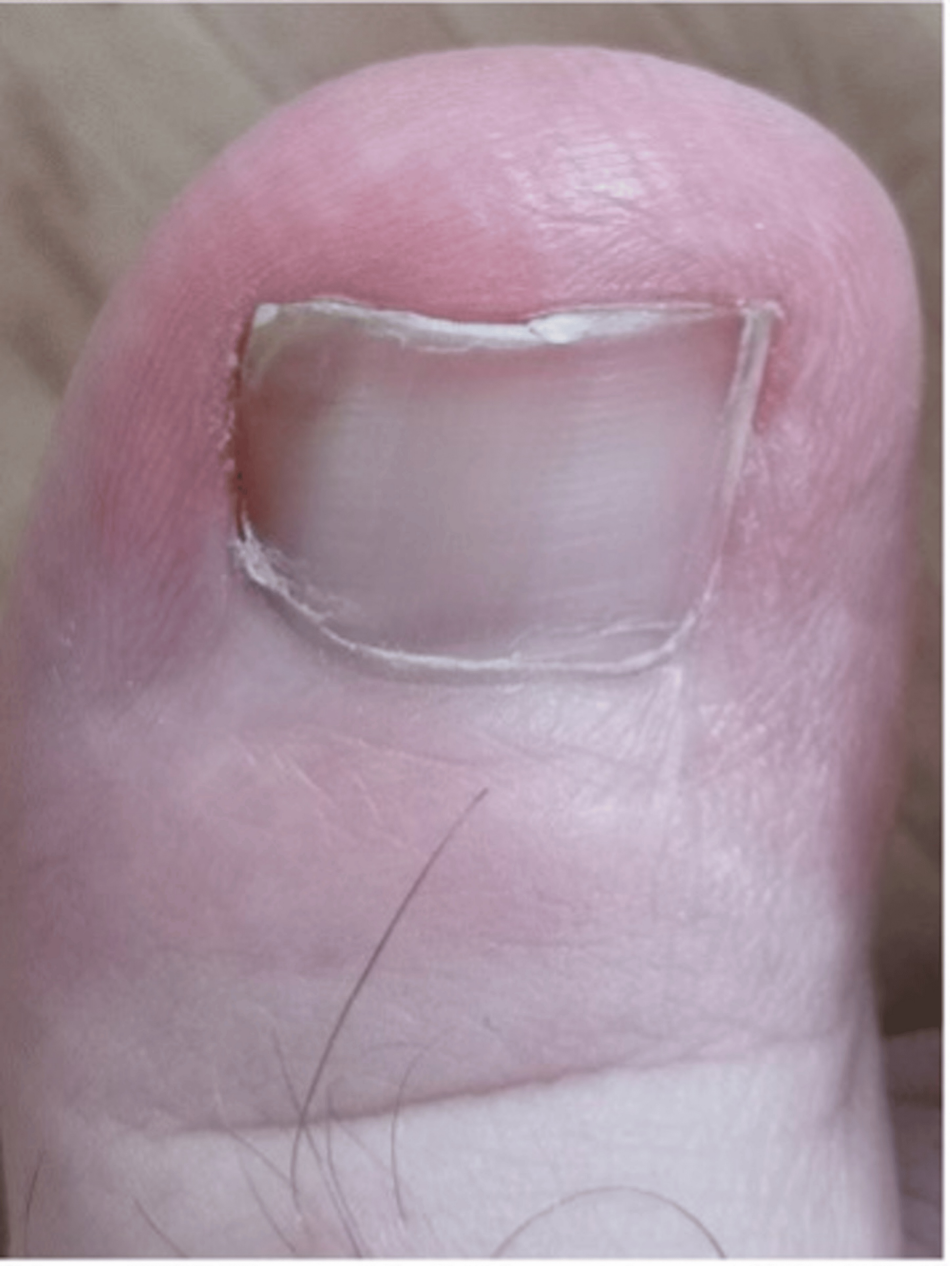
Twelve-month postoperative clinical photograph demonstrating a well-healed lateral nail fold and maintained narrowing of the nail plate following the Winograd procedure, without evidence of recurrence

Sutures were removed at two weeks postoperatively, and the patient returned to normal footwear shortly thereafter. At the 12-month follow-up, the right hallux was fully healed with no evidence of nail regrowth or recurrence (Figure 5).

The patient reported complete resolution of symptoms, unrestricted return to daily activities, and high satisfaction with the surgical outcome.

## Discussion

Currently, there are no universally accepted national guidelines that define the optimal surgical approach for onychocryptosis in the United Kingdom. This case illustrates the successful escalation of surgical management in a patient with recurrent onychocryptosis following a failed partial nail avulsion with chemical matrixectomy. Despite appropriate initial treatment and good compliance postoperatively, the patient developed nail spicule regrowth with progressive hypergranulation tissue and persistent drainage that was consistent with severe recurrent disease [6]. This clinical course highlights a recognized limitation of non-incisional nail surgery in selected patients and supports the consideration of incisional techniques when recurrence occurs. Partial nail avulsion with phenol matrixectomy is commonly used. It is relatively simple, has low morbidity, and generally produces favourable outcomes. Recurrence following phenolization has been reported in the literature and is often attributed to the incomplete destruction of the nail matrix or failure to address hypergranulation tissue [7]. In this case, the recurrence was localized to the lateral nail sulcus and associated with significant soft tissue proliferation, suggesting that chemical ablation alone was insufficient to prevent ongoing pathology. The decision to proceed with an incisional nail surgery technique was based on several patient-specific factors, including disease severity, failure of prior intervention, localization of pathology, and absence of medical contraindications. The Winograd procedure allows for the excision of both the affected nail matrix and associated hypertrophic soft tissue, thereby addressing structural contributors to recurrence that may persist following non-incisional approaches [8]. The Zadik procedure was also considered; however, the patient elected a partial incisional avulsion for cosmetic considerations. Notably, there is limited evidence comparing the efficacy of the Zadik procedure with that of the Winograd procedure [9].

Recent reports demonstrate high patient satisfaction rates, acceptable cosmetic outcomes, and low recurrence when the procedure is performed with meticulous technique and appropriate patient selection [4,10]. This case demonstrates how the Winograd procedure resulted in uncomplicated healing, an early return to normal footwear, and complete symptom resolution at the 12-month follow-up. These findings are consistent with reported outcomes demonstrating that incisional nail surgery can provide durable long-term resolution in cases of recurrent or severe onychocryptosis [3,6,11]. No postoperative complications, neurovascular compromise, or infection was observed, further supporting the safety of this approach in a controlled surgical setting. Persistent or recurrent periungual pathology should also prompt clinicians to consider alternative or coexisting diagnoses particularly when healing is delayed or clinical features are atypical. Although the presentation in this case was consistent with recurrent onychocryptosis, maintaining a broad differential diagnosis remains essential. The differential diagnosis of onychocryptosis includes conditions that produce periungual inflammation or nail plate deformity, such as paronychia, pyogenic granuloma, subungual exostosis, onychomycosis, and periungual verruca. Rarely, nail unit malignancies such as subungual melanoma should also be considered in chronic or atypical cases [12-17]. Histopathological assessment should be considered when clinical uncertainty exists [18].

## Conclusions

This case highlights the importance of appropriate surgical escalation in the management of recurrent onychocryptosis. When conservative and non-incisional treatments fail, incisional nail surgery using the Winograd procedure may offer a definitive treatment option in appropriately selected patients. The patient successfully underwent a Winograd procedure following a failed partial nail avulsion with chemical matrixectomy. The recovery was uneventful with the patient highly satisfied and pain-free 12 months postoperatively.
